# The association between FTO rs9939609 gene polymorphism and anthropometric indices in adults

**DOI:** 10.1186/s40101-020-00224-y

**Published:** 2020-05-12

**Authors:** Mahsa Mehrdad, Majid Fardaei, Mohammad Fararouei, Mohammad Hassan Eftekhari

**Affiliations:** 1 Department of Clinical Nutrition, School of Nutrition & Food Sciences, Shiraz University of Medical Sciences, Shiraz, Iran; 2 Department of Medical Genetic, School of Medicine, Shiraz University of Medical Sciences, Shiraz, Iran; 3 HIV/AIDs Research Center, Shiraz University of Medical Sciences, Shiraz, Iran

**Keywords:** Adiposity, Genotype, Overweight, Body mass index, Body fat distribution

## Abstract

**Background:**

Fat mass and obesity-associated gene (*FTO*) is the most studied obesity-related gene up to date. We aimed to assess anthropometric indices in carriers of *FTO* rs9939609 polymorphism with overweight across Iranian population (Shiraz) to find out the associations of this polymorphism with obesity indices.

**Methods:**

This was a cross-sectional study conducted on 198 overweight healthy adults aged 20-45 years old. We assessed the body composition of the participants using bioelectrical impedance analyzer. In addition, we measured the waist circumference (WC) and hip circumference (HC). Waist to hip ratio (WHR) and waist to height ratio (WHtR) were also calculated by equations. The participants’ genotype was determined by ARMS-PCR. Also, data analysis was performed using SPSS software version 20 and R software version 3.6.2.

**Results:**

The mean values of body mass index (BMI) and age of the participants were 26.93 ± 1.13 kg/m^2^ and 33.33 ± 6.35 years old, respectively. Homozygous carriers of A-allele had significantly higher values for BMI (0.60 kg/m^2^, *p* = 0.026), WHR (0.04 unit, *p* = 0.003), and WHtR (0.02 unit, *p* = 0.030) than the homozygous carriers of T-allele. Individuals with AA genotype had greater WC (2.66 cm, *p* = 0.042, and 4.03 cm, *p* = 0.002), fat mass (2.24 kg, *p* = 0.004, and 3.02 kg, *p* = 0.001), and trunk fat (1.53 kg, *p* = 0.001, and 2.08 kg, *p* = 0.001) compared to those with AT and TT genotypes, respectively. Interestingly, after adjustment of the confounders, significant associations were observed among rs9939609 polymorphism and BMI, Wt, WC, trunk fat percentage, WHR, and WHtR.

**Conclusions:**

A-allele of the *FTO* rs9939609 polymorphism was indicated to be associated with greater general and central obesity in adult population of Shiraz, Iran.

## Background

Obesity is a growing critical concern worldwide. The interactions between environmental and genetic factors are known as the main leading causes of obesity [1]. Among obesity-associated genetic factors, the fat mass and obesity-associated (*FTO*) gene on chromosome 16 has been recognized as one of the most studied genes to date. The *FTO* gene encodes a 2-oxoglutarate-dependent nucleic acid demethylase, which catalyzes the demethylation of 3-methylthymine in single-stranded DNA [2]. Although the *FTO* gene is expressed in most tissues, the highest expression level of this gene is in the hypothalamus [3]. Also, a recent study revealed a new function for *FTO* as a Fe^2+^-sensitive transcriptional repressor, which indicates its own ability to form an auto-regulatory loop that may be linked with the hypothalamic control of the body weight [4]. The strongest effect on obesity was found for rs9939609 polymorphism (A/T) variant in intron 1 [3].

Scuteri et al. [5] reported that several SNPs in *FTO* were associated with body mass index (BMI), total body weight, and also with hip circumference (HC). Interestingly, Zhang et al. [6] observed that several *FTO* SNPs including rs9939609 polymorphism can affect the body fatness, fat-free mass (FFM), frame size, and muscle mass. Rauhio et al. [7] reported that rs9939609 polymorphism is associated with body weight but not with body composition and fat distribution in premenopausal women. They observed that the individuals with AA genotype had a higher weight (~ 3.6 kg and ~ 10.1 kg, respectively) compared to those with AT and TT genotypes, and also, the carriers of A-allele had higher BMI and fat mass (FM) than non-carriers. Andreasen et al. [8] in a large sample of Danes found that the homozygous carriers of the A-allele had a higher weight than non-carriers, which is reflected in 1.1 kg/m^2^ for BMI and 2.3 cm for WC. As shown in the abovementioned studies, there are inconsistent results on the associations and effect sizes of rs9939609 polymorphism related to obesity indices across different populations and the FTO gene polymorphisms are associated with obesity through various mechanisms [9].

According to the abovementioned studies and the discrepancies, there is a need to replicate such studies in different geographical regions to determine the effect size of this SNP in all ethnic groups [10].

Since there are limited studies conducted in various regions of Iran, we aimed to investigate the association among *FTO* rs9939609 polymorphism and anthropometric indices, body composition, and fat distribution in healthy overweight adults in Shiraz, Iran.

## Methods

### Participants

We recruited 198 overall healthy overweight adults at Shohadaye WalFajr Health Center in Shiraz, Iran. All the participants were Persian, one of the ethnicities in Iran that is considered as a Caucasian subpopulation. The inclusion criteria were the age range between 20 and 45 years old and BMI ≥ 25.0 and ≤ 29.9; not participating in any weight management programs in the last 2 months; no weight loss greater than 5% in the last 2 months; non-emigrants; non-smokers; not using alcohol or any medications; no specific diseases such as neurological or psychiatric disorders, thyroid, liver, renal, and infectious diseases, and any other specific conditions or disorders; and also not being pregnant or lactating. All participants signed the informed consent form before participating in the study.

### Anthropometric assessment

We measured the height (barefoot) in a standardized measuring position by a non-stretch tape at the first visit. Anthropometric indices were assessed without wearing shoes and while wearing light cloths using Bioelectric Impedance Analyzer (BIA) (Tanita, Japan/BC-418). To ensure that the measurements are as precise as possible, we asked the participants to come with an empty stomach and empty bladder, consume no alcohol or large volume of other beverages within 24 h before the test, and also avoid from doing exercise for 4 h before the test. We picked out the following markers from the results of BIA assessment: weight, BMI, FM, FM percentage, FFM, trunk fat (TF), TF percentage, and total body water (TBW). We measured the waist circumference (WC) at the midpoint between the lower rib and the iliac crest using a non-stretch tape. HC was measured at the level of maximum extension of the hip using a non-stretch tape. We calculated WHR using equation of WC_(cm)_/HC_(cm)_ and WHtR by the equation of WC_(cm)_/Ht_(cm)_.

### Genotyping

Two milliliter of the peripheral venous blood samples was collected into EDTA tubes. The blood samples were stored at − 70 °C until use. Genomic DNA was extracted by DNA isolation kit (SinaPure DNA Kit, PR881612/EX6001/CinnaGen/Iran) according to the manufacturer’s recommendations. The DNA quantity was measured NanoDrop (ND1000, USA), and the extracted DNA was stored at − 20 °C until use. We determined the genotypes for *FTO* rs9939609 polymorphism (TT/AT/AA) via amplification refractory mutation system-polymerase chain reaction (ARMS-PCR). The method consists of two PCRs, which in one of them, the 3′ terminus of the reverse primer is specific for the normal allele, and in the other one, 3′ terminus is specific for the mutated allele. The sequence of the forward primer is GTGAGGAATACTAGGAGAGGAGAA, and the sequences of the reverse primers are AGAGACTATCCAAGTGCATCAGA for the normal T-allele and CAGAGACTATCCAAGTGCATCAAT for the mutated A-allele. All amplification reactions were performed in the ABI Thermocycler Veriti in a volume of 20 μl containing 10 μl of Mastermix, 1.8 μl extracted DNA, 6.8 μl of distilled water, and 0.7 μl of each forward and reverse primers. The thermal step program used the following compromised stages of one 15-min cycle at 95 °C (denaturation), 35 cycles under the following conditions: denaturation at 95 °C for 30 s, annealing at 56 °C for 30 s, extension at 72 °C for 30 s, and a final extension of 72 °C for 7 min after performing PCR; 5 μl of the product was mixed by 2 μl loading dye and then examined by electrophoresis on a 2.5% agarose gel. When a 351-bp band was obtained in both reactions, the subject from whom DNA was extracted was heterozygous (AT). When the band was obtained in only one of the two PCRs, the subject was homozygous. The homozygosity was TT if the band was obtained with the reverse primer with an A-residue in the 3′ position and AA if the T-residue was present in the 3′ position of the reverse primer.

### DNA sequencing

In order to validate the results obtained from the ARMS-PCR method, direct sequencing of the region containing the mutation was performed for one case of the heterozygote genotype and the two cases of the homozygote genotypes, which were randomly selected. The sequencing primers were above forward primer and ACAAATGTTCAAGTCACACTCAG as reverse primer.

### Statistical analysis

The normality of the data distribution was examined by Shapiro-Wilk test. The results from descriptive analyses were presented as mean ± standard deviation (SD) for performing quantitative continuous variables. Frequency tables were used to present the qualitative variables. Also, the significance level was set at 0.05. The associations of genotype with quantitative variables were tested using one-way ANOVA test. The Tukey post hoc test was performed to find any significant differences among the compared groups. The allele frequencies were estimated from the genotype information using the direct gene counting method. These statistical analyses were performed using the SPSS software (version 20). In addition, multiple linear regression was applied to assess the adjusted associations between polymorphism alleles and anthropometric indices by adjusting potentially important confounders (of gender, age, educational level, and marital status). The model-fitting procedures and checking for the normality of the residuals were conducted using R version 3.6.2. Since we were blinded to the individuals’ genotype at the visit session, no bias was expected for selecting the participants.

## Results

Regarding the FTO rs9939609 genotype, about half of the participants were AT (*n* = 99), 31% of them were TT (*n* = 60), and approximately 19% of them were homozygote for the known risk allele (*n* = 39). Minor allele frequency (MAF) was 44.7%. The observed genotype frequencies were not significantly different from what was predicted under the assumption of Hardy-Weinberg equilibrium. Details of the participants’ characteristics are shown in Table 1.

**Table 1:** Basic characteristics of the study population

Characteristics		Men	Women	Total
*N* (%)		50 (25%)	148 (75%)	198
Age (years)		34.00 ± 6.17	33.10 ± 6.41	33.33 ± 6.35
Genotype (%)	AA	10 (25.6%)	29 (74.4%)	39 (19.7%)
	AT	25 (25.3%)	74 (74.7%)	99 (50%)
	TT	15 (25.0%)	45 (75.0%)	60 (30.3%)
Height (cm)		177.16 ± 6.32	159.99 ± 5.57	164.32 ± 9.44
Weight (kg)		85.11 ± 6.85	68.89 ± 5.98	72.99 ± 9.39
Anthropometric indices				
BMI (kg/m^2^)		27.09 ± 1.22	26.87 ± 1.10	26.93 ± 1.13
Waist circumference (cm)		98.90 ± 5.16	94.56 ± 5.78	95.66 ± 5.93
Hip circumference (cm)		103.90 ± 3.73	102.46 ± 4.87	102.83 ± 4.65
Waist to hip ratio (WHR)		0.95 ± 0.05	0.92 ± 0.06	0.93 ± 0.06
Waist to height ratio (WHtR)		0.56 ± 0.03	0.59 ± 0.03	0.58 ± 0.04
Fat mass (kg)		19.19 ± 4.24	23.44 ± 3.00	22.36 ± 3.82
Fat mass percent		22.44 ± 3.94	34.01 ± 3.18	31.09 ± 6.07
Fat-free mass (kg)		65.92 ± 5.18	45.46 ± 4.51	50.63 ± 10.06
Fat-free mass percent		77.56 ± 3.94	65.99 ± 3.18	68.91 ± 6.07
Total body water (kg)		47.47 ± 6.44	30.51 ± 3.53	34.79 ± 8.61
Trunk fat (kg)		10.91 ± 2.60	12.01 ± 2.91	11.73 ± 2.27
Trunk fat percent		56.88 ± 5.32	51.05 ± 4.30	52.52 ± 5.22

Data are presented as mean ± standard deviation

*N* (%) number (frequency) of individuals, *BMI* body mass index

Table 2 shows the mean and standard deviations for the variables that were divided into three genotype groups. ANOVA test showed the differences in the variables among the three genotype groups (Table 2). There were significant differences among the three genotype groups in BMI, WC, WHR, WHtR, FM, and TF. This indicated that FTO rs9939609 polymorphism might affect these variables. The Tukey test showed the two groups had different means, and also, the mean difference was observed for each variable among groups. Significant differences among the three genotype groups were observed for WC, FM, and TF. A significant difference was observed only between the carriers AA and TT for BMI, WHR, and WHtR (Table 3).

**Table 2:** Comparison of anthropometric parameters across different genotypes of FTO rs9939609 in the study population

Items	Genotype (mean ± SD)			*p* value
	AA (*n* = 39)	AT (*n* = 99)	TT (*n* = 60)	
Total				
Age (years)	34.03 ± 5.89	32.99 ± 6.49	33.43 ± 6.46	0.683
Height (cm)	165.72 ± 8.96	163.98 ± 9.41	163.98 ± 9.84	0.591
Weight (kg)	75.24 ± 9.21	72.62 ± 9.17	72.14 ± 9.81	0.238
BMI (kg/m^2^)	27.31 ± 1.32^a^	26.91 ± 1.04^ab^	26.71 ± 1.11^b^	0.033*
WC (cm)	98.21 ± 6.60^a^	95.55 ± 5.47^b^	94.18 ± 5.74^c^	0.004*
HC (cm)	103.06 ± 4.54	102.78 ± 4.59	102.76 ± 4.87	0.176
WHR	0.95 ± 0.07^a^	0.93 ± 0.05^ab^	0.92 ± 0.05^b^	0.005*
WHtR	0.593 ± 0.044^a^	0.583 ± 0.034^ab^	0.575 ± 0.030^b^	0.039*
FM (kg)	24.40 ± 4.17^a^	22.16 ± 3.33^b^	21.38 ± 3.91^c^	< 0.001*
FM (%)	32.77 ± 6.16	31.04 ± 5.94	30.08 ± 6.08	0.098
FFM (kg)	50.84 ± 9.55	50.46 ± 10.25	50.76 ± 10.23	0.973
FFM (%)	67.23 ± 6.16	68.96 ± 5.94	69.92 ± 6.08	0.098
TBW (kg)	35.93 ± 9.02	35.02 ± 8.86	33.68 ± 7.92	0.420
TF (kg)	13.13 ± 2.40^a^	11.60 ± 1.99^b^	11.04 ± 2.27^c^	< 0.001*
TF (%)	54.09 ± 5.89	52.39 ± 4.78	51.72 ± 5.34	0.082

^a, b, and c^ Different values based on the Tukey test

^ab^The value is not significantly different from others

^*^p value < 0.05 considered as significant difference between three genotypes (one-way ANOVA results)

**Table 3:** Mean difference of variables among the genotypes

Items	Genotypes	Mean difference	*p* value
BMI (kg/m^2^)	AAwith AT	0.41	0.138
	AA with TT	0.60	0.026*
	AT with TT	0.20	0.526
WC (cm)	AAwith AT	2.66	0.042*
	AA with TT	4.03	0.002*
	AT with TT	1.37	0.320
WHR	AA with AT	0.02	0.052
	AA with TT	0.04	0.003*
	AT with TT	0.01	0.306
WHtR	AA with AT	0.01	0.300
	AA with TT	0.02	0.030*
	AT with TT	0.01	0.295
FM (kg)	AA with AT	2.24	0.004*
	AA with TT	3.02	< 0.001*
	AT with TT	0.78	0.401
TF (kg)	AA with AT	1.53	0.001*
	AA with TT	2.08	< 0.001*
	AT with TT	0.56	0.258

Post hoc Tukey test showed couple genotypes which had significant difference for each variable

*p value < 0.05 considered as significant

Multiple linear regression analysis after adjustment for age, gender, marital status, and educational level showed that this polymorphism was significantly associated with BMI, WC, WHR, and WHtR; however, it was not associated with FM. Though for FM and FFM percentages, the *p* values were marginal. In addition, the fitted models suggested significant associations of rs9939609 with Wt and TF percentages. The observed associations were significant only for the carriers of AA genotype in comparison to non-carriers. However, the association for WHtR was significant for both carriers of the AA and AT genotypes comparing to TT genotype (Table 4).

**Table 4:** Associations of anthropometric variables and rs9939609 polymorphism

Variable	Genotype	*B* coefficient	95% confidence interval	*p* value
BMI (kg/m^2^)	AT	0.24	− 0.10 to 0.59	0.168
	AA	0.58	0.15 to 1.01	0.001*
Weight (kg)	AT	0.59	− 1.36 to 2.54	0.552
	AA	2.89	0.45 to 5.34	0.020*
Waist circumference (cm)	AT	1.58	− 0.07 to 3.23	0.059
	AA	3.81	1.73 to 5.88	< 0.001*
Fat mass (kg)	AT	0.89	0.89 to 0.51	0.083
	AA	2.99	2.99 to 0.64	0.066
Fat mass percentage (%)	AT	1.04	− 0.02 to 2.09	0.053
	AA	2.73	1.41 to 4.05	0.051
FFM (kg)	AT	− 0.30	− 0.30 to 0.77	0.700
	AA	− 0.10	− 0.10 to 0.97	0.918
FFM percentage (%)	AT	− 1.04	− 2.09 to 0.02	0.053
	AA	− 2.73	− 4.05 to − 1.41	0.051
Trunk fat (kg)	AT	0.62	− 0.04 to 1.28	0.066
	AA	2.07	1.23 to 2.91	0.060
Trunk fat percentage (%)	AT	0.71	− 0.75 to 2.17	0.337
	AA	2.35	0.52 to 4.18	0.012*
WHR	AT	0.01	0.00 to 0.03	0.076
	AA	0.04	0.01 to 0.06	< 0.001*
WHtR	AT	0.01	0.00 to 0.02	0.048*
	AA	0.02	0.01 to 0.03	0.005*

Adjusted for gender, age, marital status, and educational level using multiple linear regression

*p value < 0.05 considered as significant

## Discussion

We found that the risk allele (A-allele) of *FTO* rs9939609 polymorphism, particularly in the form of homozygous genotype, was associated with higher BMI, WC, FM, TF, WHR, and WHtR. However, after adjustment for age, gender, marital status, and educational level, these associations remained significant just for BMI, WC, WHR, and WHtR. In addition, this analysis showed that there were also significant associations for Wt and TF percentages. The significant association did not remain significant for FM and TF anymore, after adjusting the confounders. These findings indicate that the risk allele might affect adiposity, specifically central obesity. The small difference observed in the effect size of these polymorphism alleles might result in misinterpretation by clinicians as a low clinical importance. It should be noted that these differences were caused by the effect of one SNP. Several SNPs in combination might lead to greater effects, which clinically makes the outcomes more critical. These findings showed that this polymorphism might affect the body fat distribution in addition to general fatness.

The risk allele frequency observed in this population (44.7%) was close to the frequency in Europeans (45.0%) and inconsistent with the frequency of East Asians (12.6%) [11]. Accordingly, it is an important finding since it reflects the higher frequency of the individuals with risk allele, and subsequently higher frequency of the individuals with the homozygous genotype for risk allele in this population of Iranians. It can be concluded that this polymorphism is a considerable risk factor for obesity and its comorbidities among the Iranian population, and more research should be conducted in this field, and the clinicians should consider its effects on the prevention and treatment.

There are several pathways suggested for the exact underlying mechanism of the effect of *FTO* on obesity. Remarkably, *FTO* is the first recognized demethylase, which reverses N^6^-methyladenosine (m^6^A) in RNA methylation [9]. It has been proposed that m^6^A regulates adipogenesis through mediating the mRNA splicing [12]. Another study suggested that *FTO* can regulate adipogenesis through modulation of mitotic clonal expansion [13]. Recently, Wu et al. [14] found that *FTO* promoted adipogenesis of 3 T3-L1 pre-adipocytes through regulating the cell cycle procession in an m^6^A-YTHDF2 dependent way. Tews et al. [15] observed that *FTO* deficiency induces browning of the white adipose tissue including enhanced UCP-1 expression and mitochondrial uncoupling. Also, they claimed that *FTO* can raise the susceptibility to obesity and overweight by inhibiting white adipose tissue browning [16]. Also, another study found that FTO gene expression may be associated with a change in the percentage of skeletal muscle [17]. Despite all these suggestions, the functional role of the *FTO* gene and its variants is not fully understood yet.

In accordance with our findings, Frayling et al. demonstrated that the homozygous carriers of A-allele of rs9939609 polymorphism are the significant risk factors for obesity as they weighed 3 kg heavier than the homozygotes for the T-allele [3]. Macekova et al. [18] revealed that in a population of the Roma/Gypsy individuals, WC was larger in the participants with AA genotype compared to those with TT genotype. They also observed a strong association between A-allele and BMI. Ghafarian-Alipour et al. [19] observed that women who carried the risk allele of rs9939609 polymorphism had a significantly higher weight and WC than non-carriers. Fawwad et al. [17] observed the increased central obesity with A-allele in Karachi, Pakistan, and Vasan et al. [20] observed that WHR was greater among adolescents with AA genotype than TT genotype. Despite the differences in effect size and age groups, these studies showed that, in line with our study, this variant could affect the body fat distribution and is associated with central obesity in addition to general obesity.

Inconsistent with our results, Rauhio et al. [7] in their study conducted on obese women observed that rs9939609 polymorphism was not associated with fat distribution and might only affect general obesity. In their study, differences between the genotypes for WC and HC were not statistically significant; however, these differences were clinically significant, since we observed that the mean WC in their study for AA genotype was approximately 3 and 7 cm greater than for AT and TT genotypes, respectively. These differences are greater than those we observed for WC and are also clinically important for one SNP. Interestingly, in another study performed in Tabriz by Majdi et al. [21], no significant difference was found for BMI among different genotypes. It might be due to the different ethnicities of Tabriz and Shiraz, despite belonging to one country (Iran), since the major ethnicity in Tabriz is Turkish and that in Shiraz is Persian, considered as a subpopulation of Caucasians. Likewise, Solak et al. [22] observed no correlations between rs9939609 polymorphism and obesity-related parameters in the population of Turkey whose ethnicity was Turkish. Their findings were different from ours in the genotype frequencies, MAF, and the impact on obesity as BMI. In their study, MAF of 11.11% was observed in the healthy individuals, with a frequency of 79.0, 19.7, and 0.4% for TT, AT, and AA genotypes, respectively. The reason for the discrepancy might be due to the differences among the geographical regions, which might affect the impact of the polymorphisms [22]. In addition, it should be noted that sample size can play a role in the differences observed in the outcomes of various studies.

The frequency that we observed was near the MAF; Mojaver et al. reported for the healthy individuals (42%) in Isfahan. The frequencies of TT, AT, and AA genotypes were 32, 52, and 16% in their populations, respectively [23]. This small difference in the frequencies between their study and ours might be due to the geographical variations, since they recruited the individuals from Isfahan and our participants were from Shiraz. These differences necessitate the replication of the polymorphism studies across different geographic regions.

The strength of our study was the recruitment of individuals with a narrow range of BMI (26.93 ± 1.13 kg/m^2^). Therefore, we could not attribute the differences of WC, WHR, WHtR, and TF percentages to being overweight. Further, we could attribute these differences to the type of alleles an individual carries. Another strength of our study was recruiting one ethnicity in the city, which might result in approximate homogeneity for the genetic make-up of the participants, and subsequently valid results.

We recommend the replication of these types of studies across different regions of Iran, and also with other variants of FTO to better understand the effect of this gene on obesity and its management.

## Conclusions

We observed that the risk allele of FTO rs9939609 was associated with greater central and general obesity indices in adults with overweight in Shiraz. This SNP might affect the body fat distribution in addition to general fatness in this population. Further studies are needed to discover the true mechanisms underlying the effects of these genetic variants on obesity.

## Data Availability

Not applicable

## Abbreviations

BIA: Bioelectric Impedance Analyzer
BMI: Body mass index
Wt: Weight
WC: Waist circumference
HC: Hip circumference
WHR: Waist to hip ratio
WHtR: Waist to height ratio
